# Ein mathematisches Modell zur Schätzung der Dunkelziffer von SARS-CoV-2-Infektionen in der Frühphase der Pandemie am Beispiel Deutschland und Italien

**DOI:** 10.1007/s00103-021-03384-z

**Published:** 2021-07-23

**Authors:** Jochen Fiedler, Christian P. Moritz, Sascha Feth, Michael Speckert, Klaus Dreßler, Anita Schöbel

**Affiliations:** 1 Fraunhofer Institut für Techno- und Wirtschaftsmathematik (ITWM), Fraunhofer-Platz 1, 67663 Kaiserslautern, Deutschland; 2 University hospital CHU Saint-Etienne, Saint-Étienne, Frankreich; 3 Institut NeuroMyoGène INSERM U1217/CNRS UMR 5310, Université de Lyon, Lyon, Frankreich; 4 Medicine Faculty, Université Jean Monnet, Saint-Étienne, Frankreich

**Keywords:** COVID-19, Epidemiologische Modellierung, Prävalenzschätzung, Fallsterblichkeit, COVID-19, Epidemic modelling, Prevalence estimation, Case fatality rate

## Abstract

**Hintergrund:**

Gerade in der Frühphase einer Pandemie ist es schwierig, verlässliche Zahlen über deren Ausbreitung zu erhalten. Die derzeitige COVID-19-Pandemie und das damit verbundene umfassende, aber nicht vollständige Datenmonitoring bieten die Möglichkeit, die Dunkelziffer der nicht erfassten Fälle zu schätzen.

**Ziel:**

Vorstellung eines einfachen mathematischen Modells, welches eine frühzeitige Abschätzung der Zahl nichtregistrierter Fälle (Dunkelziffer) ermöglicht.

**Material und Methoden:**

Es werden die Prävalenzen der gemeldeten Infektionen in verschiedenen Altersgruppen mit Kennzahlen der altersabhängigen Kontaktzahlen kombiniert. Daraus wird für jede Altersgruppe eine korrigierte Prävalenz abgeleitet, mit der dann die Dunkelziffer geschätzt werden kann.

**Ergebnisse:**

Unser Modell berechnet für Mitte April 2020 in Deutschland insgesamt 2,8-mal so viele Infektionen wie die Zahl der registrierten Infektionen (Fälle). Für Italien ergibt sich Mitte April 2020 ein Faktor von 8,3. Die daraus abgeleiteten Fallsterblichkeiten betragen 0,98 % für Deutschland und 1,51 % für Italien, welche deutlich näher zusammenliegen als die rein aus den zu dem Zeitpunkt vorhandenen Meldezahlen abgeleiteten Fallsterblichkeiten von 2,7 % und 12,6 %.

**Diskussion:**

Die aus dem Modell abgeleitete Dunkelziffer kann die unterschiedlichen Beobachtungen in den Fallsterblichkeiten und der Zustände in der Frühphase der COVID-19-Pandemie in Deutschland und Italien zu einem großen Teil erklären. Das Modell ist einfach, schnell und robust implementierbar und kann gut darauf reagieren, wenn die Meldezahlen hinsichtlich der Altersstruktur nicht repräsentativ für die Bevölkerung sind. Wir empfehlen, dieses Modell für eine effiziente und frühzeitige Schätzung nichtgemeldeter Fallzahlen bei zukünftigen Epidemien und Pandemien in Betracht zu ziehen.

## Abstract (en)

**Background:**

Especially in the early phase, it is difficult to obtain reliable figures on the spread of a pandemic. The effects of the COVID-19 pandemic and the associated comprehensive but incomplete data monitoring provide a strong reason to estimate the number of unreported cases.

**Aim:**

The aim of this paper is to present a simple mathematical model that allows early estimation of the number of unregistered cases (underreporting).

**Material and methods:**

Prevalences of reported infections in different age groups are combined with additional assumptions on relative contact rates. From this, a corrected prevalence is derived for each age group, which can then be used to estimate the number of unreported cases.

**Results:**

Our model derives for Germany in mid-April 2020 about 2.8 times more total infections than registered cases. For Italy, the model results in a factor of 8.3. The case mortalities derived from this are 0.98% for Germany and 1.51% for Italy, which are much closer together than the case mortalities of 2.7% and 12.6% derived purely from the number of reports available at that time.

**Conclusion:**

The number of unreported SARS-CoV-2-infected cases derived from the model can largely explain the difference in observations in case mortalities and of conditions in the early phase of the COVID-19 pandemic in Germany and Italy. The model is simple, fast, and robust to implement, and can respond well when the reporting numbers are not representative of the population in terms of age structure. We suggest considering this model for efficient and early estimations of unreported case numbers in future epidemics and pandemics.

## Hintergrund und Hypothese

COVID-19 ist eine hochinfektiöse Krankheit, die durch das neuartige Coronavirus SARS-CoV‑2 verursacht wird. Die andauernde COVID-19-Pandemie wurde von der Weltgesundheitsorganisation (WHO) zur gesundheitlichen Notlage internationaler Tragweite erklärt [1, 2]. Die Pandemie wird weltweit mit mehr als 3,5 Mio. Todesfällen (Stand: 08.06.2021) in Verbindung gebracht und verursacht gesellschaftliche Probleme durch schwerwiegende Konsequenzen für Gesundheitswesen, Sozialwesen und Wirtschaft.

Die Symptome der Krankheit sind unspezifisch und stark heterogen [3], weshalb sie kaum zur Diagnose herangezogen werden können. Stattdessen erfolgt die Diagnostik mithilfe molekularer Tests über die reverse Transkriptionspolymerasekettenreaktion (RT-PCR), bei der das Virus über seine RNA identifiziert wird [4]. Die kontinuierlich veröffentlichten offiziellen COVID-19-Fallzahlen werden größtenteils über diesen Test ermittelt. Es wird jedoch angenommen, dass diese Anzahl von SARS-CoV-2-Fällen deutlich unterschätzt wird, hauptsächlich aufgrund nicht gemeldeter (weil nicht entdeckter) Fälle durch asymptomatische Krankheitsformen und/oder begrenzter diagnostischer Kapazitäten [5, 6]. Die resultierende Dunkelziffer schwankt hypothetisch stark zwischen Ländern in Abhängigkeit von lokalen Teststrategien und -kapazitäten.

Infolgedessen ist die Gesamtzahl der infizierten Fälle als der wesentliche Schlüsselwert für das Verständnis des Krankheitsfortschrittes derzeit nicht bekannt. Insbesondere zu Beginn einer Pandemie ist dies aber meistens der Fall. Die Schätzung der Gesamtzahl infizierter Fälle ist wichtig, um 1) die Ausbreitung der Pandemie zu verfolgen, 2) die Sterblichkeitsrate zu berechnen und 3) die fortschreitende Ausbreitung einer potenziellen Herdenimmunität abzuschätzen. Somit wirkt sich die Schätzung der Dunkelziffer auch auf die mathematische Modellierung aus, wie in dem Artikel von Priesemann et al. zur Rolle epidemiologischer Modelle bei der Beschreibung des Ausbruchsgeschehens in diesem Themenheft genauer untersucht wird.

Daher sind Methoden erforderlich, mit deren Hilfe die Anzahl unentdeckter Fälle abgeschätzt werden kann. Beispielsweise wurden serologische Antikörperdetektionstests etabliert [7]. Diese sind in der frühen Phase einer Pandemie allerdings noch nicht verfügbar oder führen zu umstrittenen Schlussfolgerungen aufgrund unzureichender oder unbekannter Sensitivität und Spezifität der Test-Assays, nichttransparenter Studiendesigns [8–10] und unklarer zeitlicher Stabilität der Seroprävalenz [11]. Als komplementäre Methode zu Antikörper-Assays schlagen wir ein mathematisches Modell vor, das insbesondere in der Frühphase einer Pandemie angewendet werden kann. Das Modell haben wir im Rahmen der COVID-19-Pandemie entwickelt und erprobt, allerdings ist seine Anwendbarkeit nicht auf diese beschränkt. Das Modell macht starken Gebrauch von nach Altersgruppen aufgelösten Daten, anders als andere mathematische Modelle zur direkten Schätzung der tatsächlichen Infektionszahlen [12, 13], und stellt somit einen ergänzenden Ansatz dar.

Unsere Motivation, das Modell zu entwickeln, war die Hypothese, dass sich die Dunkelziffern zwischen den Ländern in der Frühphase einer Pandemie stark voneinander unterscheiden. Wir zeigen das für COVID-19 durch einen Vergleich der Länder Deutschland und Italien, die sich als gute Modellländer erweisen. Diese Hypothese möchten wir im Folgenden durch 2 Sachverhalte erläutern.

### Sachverhalt 1.

**Figure 1:**
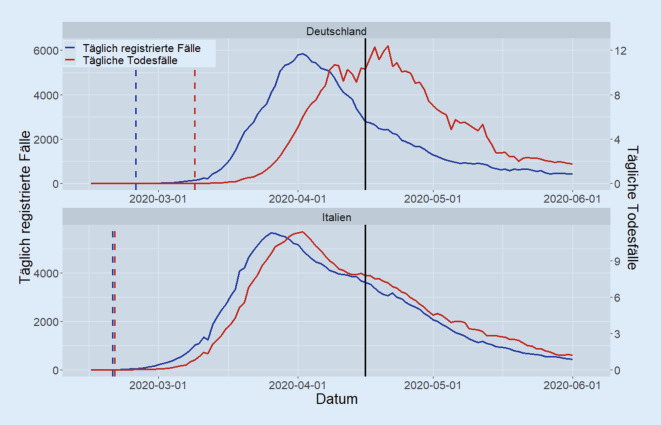

In Deutschland stiegen die gemeldeten Infektionszahlen ab dem 25.02.2020 täglich an, während die Anzahl der COVID-19-Todesfälle ab dem 09.03.2020 kontinuierlich zu steigen begannen. Im Gegensatz dazu sehen wir in Italien ab dem 21.02.2020 sowohl den beginnenden Anstieg der Infektionszahlen als auch den Beginn des Anstiegs der Anzahl an COVID-19-Todesfällen (Abb. 1). Letzteres stellt offensichtlich ein Artefakt dar, da Menschen nicht am Tag des Ausbruchs ihrer Infektion sterben. Somit spiegelt sich hier vielmehr wider, dass der Ausbruch der SARS-CoV-2-Infektion in Deutschland relativ gesehen früher als in Italien registriert wurde. In Italien entstand dadurch eine zeitliche Verzögerung, in der nicht gemeldete COVID-19-Fälle ohne Kontrolle zunehmen konnten.

### Sachverhalt 2.

Die Fallsterblichkeit in der Frühphase von COVID-19 beträgt für Deutschland 2,7 % und für Italien 12,6 % (Stichtag: 16.04.2020, inklusive abgeschlossener und offener Fälle). Wir gehen davon aus, dass der Faktor 4,7 zwischen diesen beiden gemeldeten Fallsterblichkeiten nicht auf Unterschieden in den Sterblichkeitsraten der Länder beruht, sondern auf unvollständigen Kenntnissen über die tatsächlichen Fallzahlen. Das heißt, die Dunkelziffern in Deutschland und Italien sind unterschiedlich.

Eine Begründung für diese unterschiedlichen Dunkelziffern liegt unter anderem in der zeitlichen Entwicklung der Pandemie. Italien war als erstes europäisches Land stark von der Pandemie betroffen, was sich in einem rapiden Anstieg von Fällen äußerte, die intensivmedizinische Betreuung brauchten. Da es gerade zu Beginn der Pandemie noch an systematischer Teststruktur fehlte und die Gesundheitsbehörden durch den plötzlichen Anstieg stark belastet waren, konnten nicht genügend Infektionsketten hinreichend verfolgt werden. Mildere Fälle wurden möglicherweise überproportional nicht getestet und blieben unentdeckt. Da die Wahrscheinlichkeit schwerwiegender Symptome mit dem Alter zunimmt [3], unterscheidet sich auch die Altersstruktur innerhalb aller gemeldeten Fälle und die älteren Patienten sind überrepräsentiert.

Unser *Ziel* besteht darin, mittels eines einfachen mathematischen Modells die Dunkelziffern in Deutschland und Italien zu schätzen und dadurch die anhand der Zahlen beobachteten unterschiedlichen Fallsterblichkeiten zumindest teilweise zu erklären.

Wir formulieren ein mathematisches Modell, das die Prävalenzen (Grad der Verbreitung einer andauernden oder zurückliegenden SARS-CoV-2-Infektion in der Bevölkerung) verschiedener Altersgruppen aus den gemeldeten Infektionen mit den Prävalenzen vergleicht, die sich aufgrund von unterschiedlicher Mobilität und sozialem Verhalten ergeben sollten. Von diesen korrigierten Prävalenzen erwarten wir, dass die daraus ermittelten Fallsterblichkeiten weniger Unterschiede zwischen z. B. Italien und Deutschland zeigen. Wir fassen das Vorgehen zur Bestimmung der Dunkelziffer zunächst kurz zusammen und geben später Details, die wir anhand der verwendeten Daten illustrieren.

## Methoden

### Verfahren zur Schätzung der Dunkelziffer

Weil nicht jede Infektion zuverlässig erkannt und gemeldet wird, ist die tatsächliche Prävalenz unbekannt. Bestimmt man die Prävalenz auf Basis der gemeldeten Infektionszahlen, so liegt damit eine untere Abschätzung der wahren Prävalenz vor. Diese untere Abschätzung wollen wir im Weiteren als *gemeldete Prävalenz* bezeichnen.

Um die Lücke zwischen wahrer und gemeldeter Prävalenz rechnerisch zu schließen, bestimmen wir die Unterschiede in den Infektionspotenzialen für die verschiedenen Altersgruppen und vergleichen diese mit den Unterschieden in den gemeldeten Prävalenzen. Dazu muss das altersabhängige Infektionspotenzial (bedingt durch Anzahl sozialer Kontakte, Empfänglichkeit für das Virus) aus den Prävalenzen herausgerechnet werden.

#### Schritt 1: Normalisieren mit Infektionspotenzial.

**Figure 2:**
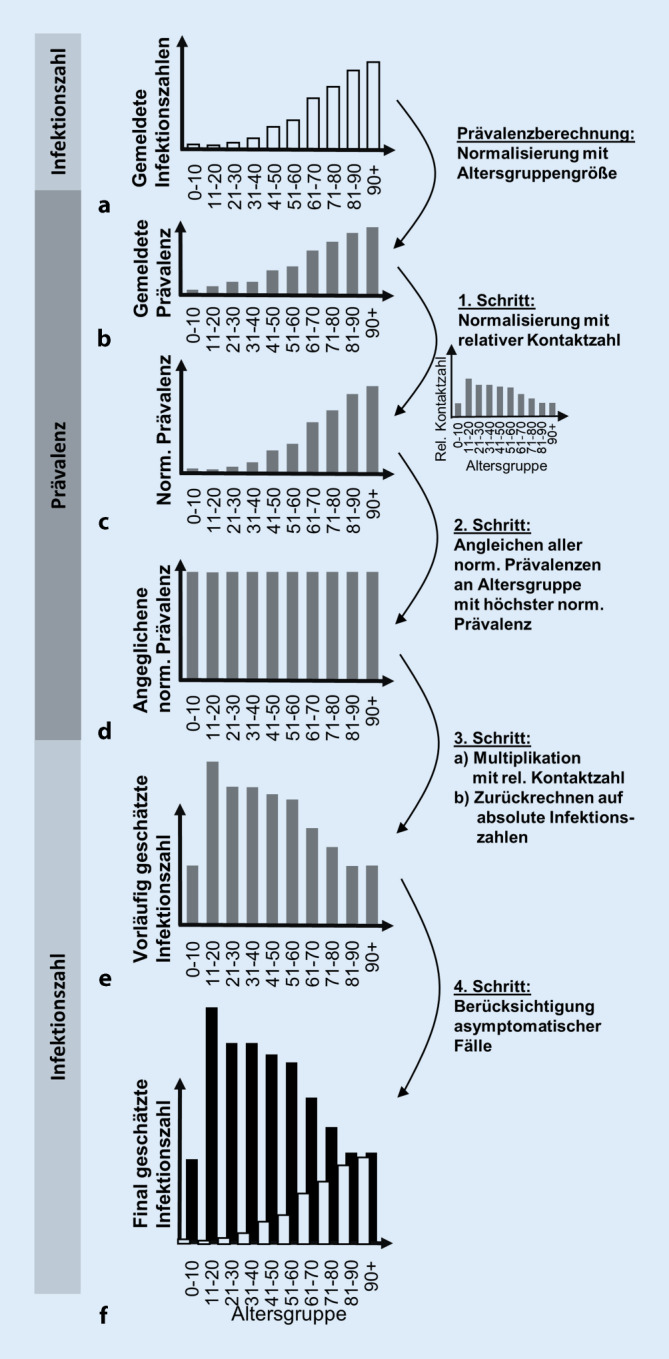

Im ersten Schritt schauen wir auf die gemeldeten Infektionszahlen der verschiedenen Altersgruppen in einer gegebenen Bevölkerung (Abb. 2a), zum Beispiel in Italien oder Deutschland. Die zugehörigen gemeldeten Prävalenzen (Abb. 2b) werden mit den relativen Kontaktzahlen (also dem Infektionspotenzial) normalisiert, da wir davon ausgehen, dass die wahren Prävalenzen proportional zu den relativen Kontaktzahlen sind. Die so erhaltenen *normalisierten* (gemeldeten) Prävalenzen (Abb. 2c) werden dann miteinander verglichen.

#### Schritt 2: Hochrechnung auf Referenzaltersgruppe.

Im zweiten Schritt wird diejenige Altersgruppe mit der höchsten normalisierten Prävalenz als Referenz gewählt. Die normalisierten Prävalenzen der übrigen Altersgruppen werden auf diese Referenz hochgerechnet (Abb. 2d). Dieser Schritt entspricht der Annahme, dass die relativen Unterschiede in den tatsächlichen Prävalenzen proportional zu den Unterschieden in den Infektionspotenzialen sein sollten.

#### Schritt 3: Infektionspotenzial wieder berücksichtigen.

Der dritte Schritt umfasst die Hochrechnung der normalisierten Prävalenzen auf Schätzungen der tatsächlichen Prävalenzen, indem mit den relativen Kontaktzahlen multipliziert wird. Wir erhalten eine vorläufige Schätzung der wahren Prävalenz. Multipliziert mit der Größe der Altersgruppen erhalten wir eine *vorläufige Schätzung der wahren Infektionszahlen *(Abb. 2e).

#### Schritt 4: Asymptomatische Fälle berücksichtigen.

In diesem Schritt berücksichtigen wir zusätzlich explizit die Anzahl der asymptomatischen Fälle. Wir gehen dazu davon aus, dass von diesen Fällen in der Frühphase einer Pandemie nur vernachlässigbar viele erfasst werden, da diese nicht auffallen und ein systematisches Monitoring noch nicht entwickelt ist. Dieser Effekt erhöht die Schätzung der Infektionszahlen aus Schritt 3 (Abb. 2f). Wir betrachten diese Werte als *beste Schätzung der tatsächlichen, unbekannten Infektionszahlen.* Diese geschätzten Infektionszahlen nutzen wir danach, um für die Länder Italien und Deutschland Fallsterblichkeiten zu berechnen und miteinander zu vergleichen. Wir erwarten, dass damit ein Teil der Unterschiede zwischen den rein aus den gemeldeten Fallzahlen direkt berechneten Fallsterblichkeiten erklärt werden kann.

Bei der Auswahl der statistischen Werkzeuge haben wir auf unsere statistischen Kompetenzen in der Fahrzeugentwicklung^1^ zurückgegriffen. Dort werden frühe Ausfälle (z. B. bei einem niedrigen Kilometerstand) zuerst von den Vielfahrern bemerkt. Für die Hersteller ist es bei solchen Ausfällen wichtig zu erkennen, ob das Einzelfälle sind oder ob sie alle ausgelieferten Fahrzeuge einer Baureihe betreffen. Will man früh, auf Basis gemeldeter Bauteilausfälle, verlässliche Prognosen ableiten, sieht man sich mit einem sog. Missing-Data-Problem konfrontiert. Durch begründete Annahmen über die monatlichen Fahrleistungen aller Fahrzeuge im Feld kann man dort auf die Dunkelziffer schließen.

### Datengrundlage

Alle unsere Analysen beziehen sich auf die folgenden Datenquellen. Für Italien nutzen wir vornehmlich die Daten aus den Berichten des nationalen Gesundheitsinstituts *Istituto Superiore di Sanità (ISS) *[14], welche in regelmäßigen zeitlichen Abständen veröffentlicht werden und Informationen über die registrierten Infektionen in verschiedenen Altersgruppen enthalten. Für Deutschland nutzen wir hauptsächlich die Daten des Robert Koch-Instituts [15]. Einzelne Darstellungen, welche keine altersspezifischen Daten benötigen, basieren zusätzlich auf den Daten der Johns Hopkins University [16, 17]. Für die Altersverteilungen der Bevölkerungen nutzen wir die Daten von der Webseite „PopulationPyramid“ [18].

Als Referenzdatum wählen wir den 16.04.2020. Zu diesem Zeitpunkt ist in beiden Ländern schon das Maximum der ersten Welle erreicht worden (Abb. 1), sodass man die Dunkelziffer in den Frühphasen der nationalen Ausbrüche gut abschätzen kann.

### Grundannahme

Bei der Bestimmung der Dunkelziffer gehen wir davon aus, dass die Verbreitung des Virus in einer Gruppe proportional zu deren Infektionspotenzial erfolgt. Dieses Infektionspotenzial hängt von vielen verschiedenen Faktoren ab, wie bspw. der Anzahl der Kontakte und der Empfänglichkeit der Menschen in dieser Gruppe für das Virus. Gerade in der Frühphase einer Pandemie lässt sich das genaue Infektionspotenzial noch nicht gut abschätzen, da das Virus zu dem Zeitpunkt noch nicht gut verstanden wird.

Was allerdings schätzungsweise verfügbar ist, sind Kontaktzahlen innerhalb einer Gruppe. Die Anzahl der Kontakte korreliert nun stark mit den sozioökonomischen Faktoren, von denen sehr viele in der Praxis nicht hinreichend bekannt sind. Ein Faktor, für den dies bekannt und gut untersucht ist, ist das Alter (siehe [19] und [20]). Unser Modell bezieht sich auf die Frühphase einer Pandemie, in der die untersuchten und modellierten Kontaktzahlen noch nicht oder weniger stark von sanitären und sozialen Maßnahmen beeinflusst werden. Zu dem von uns betrachteten Zeitpunkt waren allerdings sowohl in Italien als auch in Deutschland solche Maßnahmen in Kraft, was sicherlich einen Einfluss auf die absoluten Kontaktzahlen hatte. Allerdings gehen wir für die beobachteten Zeiträume davon aus, dass sich die Maßnahmen relativ gesehen auf alle Altersgruppen eher gleichmäßig ausgewirkt haben. Damit nehmen wir an, dass sich die relativen Verhältnisse zwischen den Kontaktzahlen der verschiedenen Altersgruppen auch durch die Maßnahmen nicht stark geändert haben. Da die Ergebnisse unseres Modells nur von diesen relativen Unterschieden abhängen und nicht auf absolute Kontaktzahlen angewiesen sind, bedeutet diese Annahme auch keine großen Änderungen in unseren Ergebnissen.

### Anwendung des Verfahrens

Im Folgenden bezeichne $P_{\mathrm{reg},i}=F_{\mathrm{reg},i}/B_{i}$ die Prävalenz der registrierten Fälle in der Altersgruppe *i*, wobei *F*_*reg*,*i*_ die Anzahl der registrierten Fälle in Altersgruppe *i* und *B*_*i*_ die Größe der zugehörigen Bevölkerungsgruppe ist. Mit *K*_*i*_ bezeichnen wir die zugehörigen relativen Kontaktzahlen, als deren Grundlagen uns die Ergebnisse aus [19] dienen, die in Tab. 1 zu finden sind. Wir modifizieren die relativen Kontaktzahlen für Kinder unter 10 Jahren, indem wir die zugehörigen Werte aus Tab. 1 durch den Faktor 2 teilen, um die angenommene geringere Infektionsempfänglichkeit von Kindern unter 10 Jahren zu berücksichtigen [21]. Weil die Aufteilung der Altersgruppen der registrierten Fälle für Deutschland und Italien nicht mit der aus Tab. 1 übereinstimmt, interpolieren wir die relativen Kontaktzahlen linear und erhalten damit insgesamt für jede Altersgruppe *i* der registrierten Fälle eine relative Kontaktzahl *K*_*i*_.

**Table 1:** 

Altersgruppe	Relative Kontaktzahl
*00–04*	1,00
*05–09*	1,42
*10–14*	1,73
*15–19*	1,68
*20–29*	1,45
*30–39*	1,45
*40–49*	1,38
*50–59*	1,31
*60–69*	1,06
*70+*	0,81

Um zu bestimmen, welche Altersgruppe die Referenz für die Prävalenz darstellt, bilden wir im ersten Schritt die normalisierten (gemeldeten) Prävalenzen $P_{\mathrm{norm},i}=P_{\mathrm{reg},i}/K_{i}$ für alle Altersgruppen. Die normalisierte Prävalenz modelliert daher einen Zustand, in welchem in der entsprechenden Bevölkerungsgruppe jeder Mensch im Mittel genau einen Kontakt hat.

Im zweiten Schritt bestimmen wir die maximale normalisierte Prävalenz $P_{\mathrm{norm},\max }=\max _{\mathrm{i}}\left(P_{\mathrm{norm},i}\right)$ und nehmen diese dann als Referenz für alle Altersgruppen an.

Beim dritten Schritt multiplizieren wir diese einheitlichen Werte für jede Altersgruppe *i* wieder mit der zugehörigen relativen Kontaktzahl *K*_*i*_ und erhalten eine vorläufige Schätzung der wahren Prävalenz $\tilde{P}_{i}=P_{\mathrm{norm},\max }\cdot K_{i}$.

Final berücksichtigen wir im vierten Schritt, dass viele Fälle aufgrund fehlender Symptome nicht auffallen. Da wir die Rate $a_{i}=a$ dieser asymptomatischen Fälle für alle Altersgruppen als gleich annehmen, erhalten wir die (finale) Schätzung der wahren Prävalenz:

$$P_{i}=\frac{\tilde{P}_{i}}{1-a}.$$

Sobald Daten vorliegen, mit denen der Anteil der asymptomatischen Fälle besser nach Altersgruppen aufgelöst werden kann, sollte das im Modell berücksichtigt werden. Die angepassten Fallzahlen berechnen sich hieraus, indem wir wieder mit den dazugehörigen Bevölkerungszahlen multiplizieren.

Um eventuelle Verteilungsunterschiede zwischen den Geschlechtern nicht zu übersehen, führen wir dieses Verfahren für Männer und Frauen getrennt durch und erhalten somit für beide Geschlechter eine angepasste Zahl an Infektionen.

## Ergebnisse

### Normalisierte (gemeldete) Prävalenzen (Schritt 1)

Tab. 2 zeigt die gemeldeten Prävalenzen und die zugehörigen normalisierten Prävalenzen. Beispielsweise sehen wir für die Altersgruppe 80+ in Deutschland eine gemeldete Prävalenz von 0,226 %, was dividiert durch die relative Kontaktzahl von 0,81 eine normalisierte Prävalenz von 0,280 % ergibt. Die höchsten normalisierten Prävalenzen werden jeweils in den höchsten Altersgruppen beobachtet.

**Table 2:** 

Land	Altersgruppe	Männer [%]	Frauen [%]	Insgesamt [%]
Deutschland	00–04	0,027 (0,014)	0,024 (0,012)	0,026 (0,013)
	05–14	0,034 (0,016)	0,033 (0,016)	0,034 (0,016)
	15–34	0,152 (0,099)	0,181 (0,117)	0,167 (0,108)
	34–59	0,185 (0,135)	0,200 (0,147)	0,193 (0,141)
	60–79	0,157 (0,168)	0,125 (0,133)	0,140 (0,150)
	80+	0,226 (*0,280*)	0,229 (*0,282*)	0,228 (0,282)
Italien	00–09	0,022 (0,009)	0,021 (0,009)	0,022 (0,009)
	10–19	0,030 (0,018)	0,032 (0,019)	0,031 (0,018)
	20–29	0,106 (0,073)	0,146 (0,101)	0,126 (0,087)
	30–39	0,150 (0,103)	0,179 (0,123)	0,165 (0,114)
	40–49	0,196 (0,142)	0,247 (0,179)	0,222 (0,161)
	50–59	0,319 (0,244)	0,311 (0,237)	0,316 (0,241)
	60–69	0,423 (0,399)	0,234 (0,221)	0,325 (0,307)
	70–79	0,573 (0,707)	0,314 (0,388)	0,433 (0,535)
	80–89	0,865 (1,068)	0,651 (0,804)	0,736 (0,909)
	90+	1,052 (*1,299*)	1,213 (*1,498*)	1,169 (1,443)

### Referenzwerte der normalisierten Prävalenzen (Schritt 2)

Die Ergebnisse des vorherigen Abschnitts zeigen, dass in beiden Ländern jeweils die Gruppe der ältesten Menschen die höchste normalisierte Prävalenz aufweist. Damit erhalten wir als Referenzwerte die hervorgehobenen normalisierten Prävalenzen aus Tab. 2. Für Deutschland ist der Referenzwert für Männer bei 0,280 % und für Frauen bei 0,282 %, während die Referenzwerte für Männer und Frauen in Italien bei 1,299 %, bzw. 1,498 % liegen.

### Schätzung der wahren Infektionszahlen (Schritte 3 und 4)

Multiplizieren wir die bei allen Altersgruppen gleichgesetzten Referenzwerte des vorigen Abschnitts mit den zugehörigen relativen Kontaktzahlen und berücksichtigen wir die Rate der asymptomatischen Fälle, so erhalten wir die vorläufige Schätzung der wahren Prävalenzen. Für die Rate der asymptomatischen Fälle nehmen wir einen Wert von 22,2 % an, welchen wir aus den Ergebnissen der Studie von Streeck und Kollegen entnehmen [22]. Berechnen wir mithilfe der Bevölkerungszahlen die entsprechenden Infektionszahlen, so erhalten wir die Ergebnisse in Tab. 3. Dort ergibt sich der Wert für Kinder im Alter von 5–14 Jahren in Deutschland dadurch, dass wir einmal die Rechnung für Jungen und Mädchen separat durchführen und die Werte addieren. Für Mädchen sieht die Rechnung wie folgt aus: Der Referenzwert von 0,282 % wird erst mit der interpolierten relativen Kontaktzahl von 1,575 multipliziert, was eine Prävalenz von 0,444 % ergibt. Anschließend multiplizieren wir diesen Wert mit 0,75, um die halb so große Infektionsempfänglichkeit bei Kindern unter 10 Jahren zu berücksichtigen. Dieser Wert ergibt sich, da wir vereinfacht annehmen, dass die Altersgruppe 5–14 jeweils zur Hälfte aus Kindern unter und über 10 Jahren besteht. Danach multiplizieren wir weiter mit 3.637.732, der Anzahl der Mädchen in dieser Altersgruppe, und teilen durch 0,77, was die asymptomatischen Fälle berücksichtigt, und erhalten als finale Schätzung für die Zahl der infizierten Mädchen in dieser Altersgruppe 15.580. Addieren wir darauf die Fälle für die Jungen, welche auf gleiche Weise berechnet werden, so erhalten wir den Wert von 31.923 aus Tab. 3.

**Table 3:** 

Land	Altersgruppe	Angepasste Fallzahlen	Unregistrierte Fälle
Deutschland	00–04	7149	6111
	05–14	31.923	29.357
	15–34	105.122	73.443
	35–59	143.567	86.929
	60–79	60.207	35.019
	80+	16.727	3618
	Gesamt	364.695	234.477
Italien	00–09	55.401	54.278
	10–19	175.585	173.781
	20–29	159.600	151.863
	30–39	185.047	173.361
	40–49	228.988	208.469
	50–59	222.982	193.124
	60–69	141.306	117.266
	70–79	86.959	61.242
	80–89	53.620	26.914
	90+	12.605	2792
	Gesamt	1.322.093	1.163.090

Für Deutschland bedeuten unsere Schätzungen, dass die Zahl aller Infektionen etwa 2,8-mal so groß ist wie die Zahl der registrierten. Für Italien beträgt dieser Faktor etwa 8,3. In beiden Ländern ist die Dunkelziffer der nicht registrierten Infektionen also höher als die Zahl der registrierten Fälle.

### Vergleich der Fallsterblichkeiten

**Figure 3:**
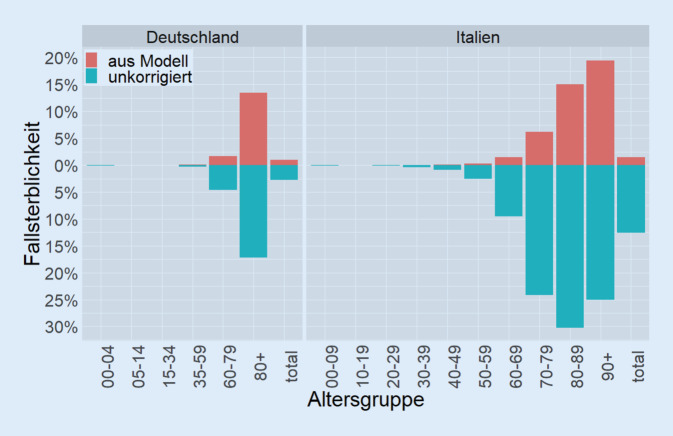

Über die geschätzten Infektionszahlen aus obigem Abschnitt lassen sich nun korrigierte Fallsterblichkeiten für alle Altersgruppen berechnen, wie in Abb. 3 dargestellt. Weil es in jeder Altersgruppe nicht entdeckte Fälle gibt, sind die korrigierten Fallsterblichkeiten für alle Altersgruppen niedriger als die unkorrigierten Fallsterblichkeiten. Wie erwartet liegen die Werte für Deutschland und Italien nun auch näher beieinander. Für die korrigierte Fallsterblichkeit über alle Altersgruppen hinweg erhalten wir 0,98 % für Deutschland, während es für Italien 1,51 % sind. Dies entspricht nun einem Faktor von 1,55. Des Weiteren sind die mittels unseres Modells geschätzten Fallsterblichkeiten für Italien monoton mit dem Lebensalter steigend, während bei den unkorrigierten Werten die Altersgruppe 90+ eine geringere Fallsterblichkeit aufweist als die Altersgruppe 80–89.

## Diskussion

Durch unser prävalenzbasiertes Modell der ersten 7–8 Wochen der Pandemie schätzten wir die Dunkelziffer und hiermit die tatsächliche Prävalenz der SARS-CoV-2-Infektionen. Diese Prävalenzen waren für Deutschland 2,8-mal und für Italien 8,3-mal höher als jene basierend auf der Anzahl gemeldeter Fälle. Demnach wären zu diesem frühen Zeitpunkt der Pandemie bereits 0,44 % der Bevölkerung Deutschlands und 2,18 % der Bevölkerung Italiens infiziert gewesen. Die Fallsterblichkeit würde demnach 0,98 % für Deutschland und 1,51 % für Italien betragen.

Um die Güte der Vorhersagen unseres mathematischen Modells zu überprüfen, nutzen wir Literaturdaten aus serologischen Untersuchungen als Referenz. Solche Antikörpertests gelten als relativ verlässlicher Nachweis für eine zurückliegende SARS-CoV-2-Infektion. Streeck und Kollegen schätzten die tatsächliche Prävalenz der SARS-CoV-2-Infektionen mittels serologischer Methoden basierend auf dem Vorhandensein spezifischer Antikörper gegen die Virusproteine [22]. Sie fanden solche Antikörper in 15,5 % der durch ein Super-spreading-Ereignis stark exponierten Population im Kreis Heinsberg. Durch dieses Ereignis ist die resultierende Prävalenz zwar nicht repräsentativ für Deutschland oder Europa, jedoch sollte der dadurch geschätzte Anteil der Dunkelziffer vergleichbar sein. Deren Antikörperstudie detektierte eine 5‑mal höhere Prävalenz als die angenommene Prävalenz basierend auf detektierten COVID-19-Fällen. Dieser Faktor liegt zwischen unseren Werten für Deutschland und Italien.

Italienische Behörden schätzten die tatsächliche Prävalenz ebenfalls mittels Antikörpertests. Nach einem landesweiten serologischen Test von 64.660 Personen wurde die Prävalenz auf 2,5 % geschätzt. Dieser Wert liegt sehr nahe an unserem Wert von 2,18 % [23].

In einem ähnlichen Test, allerdings begrenzt auf die stark exponierte Provinz Bergamo, wurden 423 Arbeiter serologisch getestet, wovon bereits 38,5 % SARS-CoV-2-Antikörper hatten. Dieser Anteil ist 26-mal so groß wie die aus den offiziell gemeldeten Fällen errechnete Prävalenz [24]. Diese starke Diskrepanz bestätigt unsere Annahme, wonach eine stärkere Überlastung der Gesundheitssysteme mit einer höheren Dunkelziffer einhergeht.

Bezüglich Fallsterblichkeit schätzten Streeck et al. [22] basierend auf ihrer Antikörperstudie einen Wert von 0,36 % (mit 95 %-Konfidenzintervall [0,29 %; 0,45 %]) für Deutschland. Dieser Wert liegt etwas unter unserer Schätzung. Die italienweite Schätzung würde mit einer Fallsterblichkeit von 2,3 % einhergehen [23], während die Studie in der Provinz Bergamo auf einen Wert von 1 % kam [24]. Die aus unserem Modell resultierende Fallsterblichkeit von 1,51 % liegt in Bereich dieser Studien.

Diese Übereinstimmung untermauert die Validität unseres Models als schnelle, effiziente und kostengünstige Methode zur Abschätzung der Dunkelziffer in einer frühen Pandemiephase. Marginale Unterschiede zwischen unseren Schätzungen und jenen aus Antikörpertests könnten auf über- oder unterschätzte Prävalenzen unseres Modells oder auf technische Grenzen der Antikörpertests (limitierte Spezifität oder nicht repräsentative Testkohorten) zurückgehen. Mögliche Abweichungen zu den Aussagen unseres Modells können bspw. auf die Unsicherheiten in den genutzten Kontaktzahlen zurückgehen, welche nie vollständig und exakt bekannt sind. Hier zeigt sich aber auch ein weiterer Vorteil unseres Modells im Vergleich zu anderen mathematischen Ausbreitungsmodellen, welche Kontaktzahlen nutzen: Unser Modell ist nur auf die relativen Unterschiede zwischen den Kontaktzahlen angewiesen und nicht auf deren absoluten Werte.

Die deutlich größere Dunkelziffer in Italien geht in unserem Modell direkt auf die Tatsache zurück, dass der Anteil an sehr alten Menschen bei den Erkrankten viel höher ist als deren Anteil an der Bevölkerung. Wir halten es für sehr plausibel, dass dies auf die Tatsache zurückzuführen ist, dass ältere Menschen im Mittel viel schwerer erkranken und damit deutlich stärker auffallen als jüngere Menschen. Diese Dunkelzifferschätzungen ermöglichen es uns, einen großen Teil des Unterschieds in den zum 16.04.2020 beobachteten Fallsterblichkeiten für Italien und Deutschland zu erklären. Der resultierende Faktor von 1,5 zwischen den Fallsterblichkeiten erscheint uns plausibler als der deutlich höhere Faktor von 4,6, der rein aus den gemeldeten Fallsterblichkeiten bestimmt wird. Die verbliebene Abweichung vom Faktor 1 lässt sich durch weitere Faktoren (wie bspw. ein lokal stark überlastetes Gesundheitssystem) erklären, die nur schwierig quantitativ zu ermitteln sind. Darüber hinaus ist die mit unserem Modell geschätzte Fallsterblichkeit für Italien monoton mit dem Lebensalter steigend, sodass die Altersgruppe 90+ das höchste Risiko aufweist, an der Krankheit zu versterben. Dies erscheint uns auch glaubwürdiger als die unkorrigierte Schätzung der Fallsterblichkeiten aus den registrierten Fallzahlen, in denen die zweithöchste Altersgruppe (80–89 Jahre) eine höhere Fallsterblichkeit aufweist als die höchste (90 Jahre und älter).

Ein großer Vorteil des Modells ist, dass es nicht viele Annahmen und Daten benötigt und nur wenige Parameter besitzt. Das ermöglicht eine einfache und gut nachvollziehbare, robuste Implementierung. Die Robustheit folgt dabei aus der Tatsache, dass sich Änderungen in den Parametern höchstens linear auf die Ergebnisse auswirken. Somit schlägt sich bei den beobachteten Prävalenzen die Korrektur der relativen Kontaktzahlen für Kinder [16] auch nur in dieser Gruppe aus: Ohne Korrektur wäre die aus unserem Modell geschätzte Fallzahl um einen Faktor 2 höher, was sich nur gering auf die Gesamtzahl nicht registrierter Fälle aller Altersgruppen zusammen auswirkt.

Unser Modell lässt sich prinzipiell immer dann bei einer Epidemie anwenden, wenn folgende Aspekte angenommen werden können: Die Ausbreitung erfolgt proportional zu den Kontaktzahlen, die Fallzahlen sind für verschiedene Altersgruppen bekannt und die relativen Kontaktzahlen für diese Altersgruppen können hinreichend gut ermittelt werden. Ändern sich die relativen Kontaktzahlen aufgrund der Auswirkung dieser Epidemie sehr stark (z. B. durch kontakteinschränkende Maßnahmen, spezifisch für ältere Menschen), so sollten die Kontaktzahlen möglichst in Echtzeit ermittelt werden. Die Annahme, dass die Ausbreitung proportional zu den Kontaktzahlen erfolgt, bedeutet, dass die Wahrscheinlichkeit einer Übertragung nicht stark vom Lebensalter abhängig ist. In unserem Fall haben wir lediglich eine geringere Übertragungswahrscheinlichkeit bei Kindern [16] dadurch korrigiert, dass wir mit modifizierten Kontaktzahlen rechnen. Dies lässt sich auch prinzipiell bei anderen Epidemien anwenden: Ist bekannt, dass verschiedene Altersgruppen das Virus aufgrund eben ihres Alters unterschiedlich stark weitergeben, dann lassen sich so wieder modifizierte Kontaktzahlen bestimmen, welche dann von unserem Modell genutzt werden können, was eine hohe Anpassungsfähigkeit bedeutet.

## Schlussfolgerung

Unser Modell ist in der Lage, gerade in der Frühphase einer Epidemie bei einer unklaren Datenlage quantitativ gut abzuschätzen, wie stark sich ein Erreger schon verbreitet hat. Die bessere Kenntnis der Dunkelziffer kann als Handlungsgrundlage genutzt werden, um geeignete Maßnahmen einzuleiten.

## Footnotes

1 https://www.itwm.fraunhofer.de/de/abteilungen/mf.html.
